# Changes in cardiac arrest patients’ temperature management after the 2013 “*TTM*” trial: results from an international survey

**DOI:** 10.1186/s13613-015-0104-6

**Published:** 2016-01-12

**Authors:** Nicolas Deye, François Vincent, Philippe Michel, Stephan Ehrmann, Daniel da Silva, Michael Piagnerelli, Antoine Kimmoun, Olfa Hamzaoui, Jean-Claude Lacherade, Bernard de Jonghe, Florence Brouard, Corinne Audoin, Xavier Monnet, Pierre-François Laterre, For the SRLF Trial Group

**Affiliations:** Réanimation Médicale et Toxicologique, Unité Inserm U942, Centre Hospitalier Universitaire Lariboisière, Assistance Publique des Hôpitaux de Paris, 2, rue Ambroise Paré, 75010 Paris, France; Réanimation Polyvalente, Groupe Hospitalier Inter-Communal Le Raincy-Montfermeil, Montfermeil, France; Réanimation Polyvalente, Centre Hospitalier Régional René Dubost, Pontoise, France; Réanimation Polyvalente, Centre Hospitalier Régional Universitaire, Tours, France; Réanimation, Centre Hospitalier Delafontaine, Saint-Denis, France; Department of Intensive Care Experimental Medicine Laboratory, Centre Hospitalier Universitaire, Charleroi, Belgium; Réanimation Médicale, Centre Hospitalier Universitaire de Nancy Brabois, Vandoeuvre-les-Nancy, France; Réanimation Polyvalente, Hôpital Antoine Béclère, APHP, Clamart, France; Réanimation Polyvalente, Centre Hospitalier Départemental Les Oudairies, La Roche-Sur-Yon, France; Réanimation Médicale, Centre Hospitalier Inter-Communal, Poissy, France; Clinique des Cèdres-Cornebarrieu, Blagnac, France; Réanimation Médicale, Centre Hospitalier Universitaire Paris-Sud, APHP, Kremlin-Bicêtre, France; Medical-surgical intensive care unit, Saint Luc University Hospital, Université Catholique de Louvain, Brussels, Belgium

**Keywords:** Survey, Therapeutic hypothermia, Targeted temperature management, Cooling, Temperature, Heart arrest

## Abstract

**Background:**

Therapeutic hypothermia (TH between 32 and 34 °C) was recommended until recently in unconscious successfully resuscitated cardiac arrest (CA) patients, especially after initial shockable rhythm. A randomized controlled trial published in 2013 observed similar outcome between a 36 °C-targeted temperature management (TTM) and a 33 °C-TTM. The main aim of our study was to assess the impact of this publication on physicians regarding their TTM practical changes.

**Methods:**

A declarative survey was performed using the webmail database of the French Intensive Care Society including 3229 physicians (from May 2014 to January 2015).

**Results:**

Five hundred and eighteen respondents from 264 ICUs in 11 countries fulfilled the survey (16 %). A specific attention was generally paid by 94 % of respondents to TTM (hyperthermia avoidance, normothermia, or TH implementation) in CA patients, whereas 6 % did not. TH between 32 and 34 °C was declared as generally maintained during 12–24 h by 78 % of respondents or during 24–48 h by 19 %. Since the TTM trial publication, 56 % of respondents declared no modification of their TTM practice, whereas 37 % declared a practical target temperature change. The new temperature targets were 35–36 °C for 23 % of respondents, and 36 °C for 14 %. The duration of overall TTM (including TH and/or normothermia) was declared as applied between 12 and 24 h in 40 %, and between 24 and 48 h in 36 %. In univariate analysis, the physicians’ TTM modification seemed related to hospital category (university versus non-university hospitals, *P* = 0.045), to TTM-specific attention paid in CA patients (*P* = 0.008), to TH durations (<12 versus 24–48 h, *P* = 0.01), and to new targets temperature (32–34 versus 35–36 °C, *P* < 0.0001).

**Conclusions:**

The TTM trial publication has induced a modification of current practices in one-third of respondents, whereas the 32–34 °C target temperature remained unchanged for 56 %. Educational actions are needed to promote knowledge translations of trial results into clinical practice. New international guidelines may contribute to this effort.

**Electronic supplementary material:**

The online version of this article (doi:10.1186/s13613-015-0104-6) contains supplementary material, which is available to authorized users.

## Background

Targeted temperature management (TTM) has been shown to improve outcome in adult patients successfully resuscitated from cardiac arrest (CA) [1–4]. However, several issues such as the optimal level of TTM or the optimal method of cooling still remained unsolved [4, 5]. The recent publication of the large TTM trial by Nielsen and co-workers seemed to answer, at least partially, those questions [6]. No main outcome differences were observed between the two evaluated levels of TTM: 33 °C (corresponding previously to therapeutic hypothermia: TH) versus 36 °C both applied during 28 h. However, this trial mostly included out-of-hospital CA patients with relatively short no-flow durations occurring from both initial non-shockable and shockable rhythms. Conversely, the European trial had previously compared a 33 °C-TTM group versus a control group without a specific temperature management protocol in patients after CA from shockable rhythm only and more prolonged no-flow durations [7]. Finally, the three main randomized trials evaluating TTM after CA seem to highlight that all TTM procedures (e.g., 36 °C-TTM and 33 °C-TTM) are similarly beneficial when compared with non-TTM regimen [1, 2, 6–8]. This issue, also reinforced by a recent meta-analysis, has been recalled by the International Liaison Committee On Resuscitation (ILCOR) experts, possibly to avoid any definitive abandon of TTM implementation after CA [9, 10]. Indeed these experts assume that the published studies do not support a treatment strategy where TTM is abandoned but support a strategy where either 33 or 36 °C-TTM remains an important component of post-CA treatment [9]. However, to the best of our knowledge, no study has to date evaluated the potential impact of these different TTM trials on the post-CA management.

Following the main studies on TH published in 2002 [1, 2], the initial surveys showed relatively low rates of TH implementation overall ranging from 13 to 28 % with variations according to countries [11–15]. After the first TH guidelines published in 2003 [16], widespread TH implementation seemed to occur progressively. Indeed, two consecutive surveys performed in Poland found that the number of intensive care units (ICUs) using TH increased threefold in the 5-year period of 2005–2010 [17, 18]. Similarly, two consecutive surveys found a major increase in TH use from 28.4 to 85.6 % between 2006 and 2010 in the United Kingdom [15, 19]. Finally, a declarative survey performed in France reported up to 98 % adherence to recommendations regarding TH [20]. As the pendulum may swing back to an adaptive “lighter” TTM or a non-TTM approach in resuscitated CA patients, we decided to conduct a survey to assess the potential impact of the recent studies published on the practical management of such patients.

## Methods

A declarative survey was performed among 3 229 physician members of the French Intensive Care Society (FICS/*SRLF*), including numerous French-speaking countries. The first email was sent on May 23, 2014, followed by eight new mailings sent monthly until January 8, 2015 (several answers per ICU were allowed). Respondents received no compensation for their participation in the survey.

A questionnaire written in French was developed by a senior intensivist experienced in CA and TTM, and implemented using SurveyMonkey^®^. The study group tested the questionnaire and worked on rephrasing and improvement. The survey was made of 74 questions, of which 19 needed mandatory answers and included those focusing on TTM practice in CA patients (see Additional file 1). Briefly, the questionnaire was divided into four parts: (1) Characteristics of the ICU and the respondent; (2) General and cardiovascular management of CA; (3) TTM in CA patients (indication for TH use, changes induced by the Nielsen trial [6], cooling methods); and (4) Prognostication in CA patients. Except for quantitative questions, responses were obtained either using a bimodal answer (“yes/no”) or a 4-point Likert scale (structured as “always/frequently/sometimes/never”).

### Statistics

Quantitative variables were expressed as mean (standard deviation) when following a Gaussian distribution or median (interquartile range 25–75 %) otherwise, and were compared using the Student t or Mann–Whitney test, respectively. Qualitative variables were expressed as frequencies (95 % confidence interval) calculated by angular transformation, and were compared using the Chi Square or Fisher exact probability test for categorical variables. Data were tested for normality using the Shapiro Normality Test. Multivariate analyses were performed using logistic regression. All tests were two-sided with 5 % significance, and performed using the R software (R package version 2.14.1.6, Vienna, Austria).

## Results

Five hundred and eighteen respondents fulfilled the survey (16 %), representing an absolute number of 264 ICUs (France: 219; Belgium: 18; Africa: 16; others from Europe: 9; Canada: 2). General characteristics of respondents and their ICUs are described in Table 1. The distribution of responses over time is depicted in Additional file 2: Figure S1.

**Table 1:** Characteristics of respondents (number of respondents: *N*) and their intensive care unit

Type of hospital (*N* = 509)	
Public university hospital	249 (49)
Public non-university hospital	228 (45)
Private hospital	27 (5)
Other	5 (1)
Country (*N* = 515)	
France	448 (87)
Other in Europe	38 (7)
Africa	25 (5)
Canada	4 (1)
ICU staff (*N* = 500)	
Attending full-time physicians, mean ± SD	7.2 ± 3.8
Resident and fellow, mean ± SD	4.8 ± 4.2
ICU beds, mean ± SD (*N* = 448)	15 ± 7
ICU activity during the last full year (2013) prior to the present study	
Overall admissions (*N* = 500)	
<300	22 (4)
300–500	125 (25)
500–800	145 (29)
>800	208 (42)
Admissions for CA (*N* = 518)	
<10	70 (14)
10–20	154 (30)
20–30	76 (15)
30–40	60 (12)
40–50	45 (9)
>50	113 (22)
TH implementation (*N* = 384)	
<10	74 (19)
10–20	120 (31)
20–30	54 (14)
30–40	45 (12)
40–50	34 (9)
>50	57 (15)
Use of a written CA procedure (*N* = 509)	221 (43)
No available CA procedure in the ICU	259 (52)
In-hospital cath lab performing coronary angiography (*N* = 518)	363 (70)
With a 24 h/24 h availability	353 (97)

Results are expressed as *n* (%) unless specified otherwise

### Targeted temperature management (TTM)

Specific attention is generally paid to temperature management (normothermia, avoiding hyperthermia, or TTM implementation) in successfully resuscitated and unconscious CA patients as declared by 94 % of respondents, mainly using TH in 89 %. The reasons why TH is not applied are depicted in Additional file 2: Figure S2. Indications of TH implementation in successfully resuscitated and unconscious CA patients are described in Table 2. Temperature monitoring after CA is mainly performed using a bladder probe as reported by 42 % of respondents, and/or an esophageal probe by 41 % of respondents (Additional file 2: Figure S3).

**Table 2:** Indications for therapeutic hypothermia implementation after successfully resuscitated and unconscious cardiac arrest patient (*N* respondents)

Out-of-hospital CA from initial shockable rhythm^a^ (*N* = 403)	
Always	258 (64)
Frequently	75 (19)
Sometimes	24 (6)
Never	29 (7)
Do not know	17 (4)
Out-of-hospital CA from initial non-shockable rhythm^b^ (*N* = 403)	
Always	134 (33)
Frequently	129 (32)
Sometimes	73 (18)
Never	51 (13)
Do not know	16 (4)
In-hospital CA from initial shockable rhythm^a^ (*N* = 399)	
Always	229 (57)
Frequently	85 (21)
Sometimes	38 (10)
Never	32 (8)
Do not know	15 (4)
In-hospital CA from initial non-shockable rhythm^b^ (*N* = 398)	
Always	117 (29)
Frequently	122 (31)
Sometimes	86 (22)
Never	60 (15)
Do not know	13 (3)

Results are expressed as *n* (%)

*CA* cardiac arrest

^a^Ventricular fibrillation/pulseless ventricular tachycardia

^b^Asystole/pulseless electrical activity

TH was declared as never induced using intravenous cold fluids by 38 % of respondents (Table 3). TH was reported to be always induced and/or maintained using basic external methods (fan and ice packs) by 34 %, whereas other methods of cooling were more rarely used. TH between 32 and 34 °C was declared as generally maintained during 12–24 h by 78 % of respondents, or during 24–48 h by 19 %. Passive rewarming was predominantly used by 66 % of respondents. The usual reported rate for active rewarming was 0.5 °C/h in 53 % and 0.3 °C/h in 25 % of the cases.

**Table 3:** Methods of cooling used for therapeutic hypothermia implementation after cardiac arrest (*N* respondents)

Cold intravenous fluid infusion to induce TH (*N* = 396)	
Always	48 (12.1)
Frequently	95 (24.0)
Sometimes	100 (25.2)
Never	152 (38.4)
Do not know	1 (0.3)
Basic surface cooling (fans, ice packs) to induce and/or maintain TH (*N* = 395)	
Always	136 (34.4)
Frequently	91 (23.0)
Sometimes	93 (23.6)
Never	74 (18.7)
Do not know	1 (0.3)
External water blanket cooling to induce and/or maintain TH (*N* = 394)	
Always	57 (14.5)
Frequently	34 (8.6)
Sometimes	26 (6.6)
Never	274 (69.5)
Do not know	3 (0.8)
External air blanket cooling to induce and/or maintain TH (*N* = 396)	
Always	28 (7.1)
Frequently	38 (9.6)
Sometimes	59 (14.9)
Never	269 (67.9)
Do not know	2 (0.5)
External advanced surface cooling gel pads to induce and/or maintain TH (*N* = 399)	
Always	10 (2.5)
Frequently	30 (7.5)
Sometimes	25 (6.3)
Never	328 (82.2)
Do not know	6 (1.5)
Intravascular device to induce and/or maintain TH (*N* = 395)	
Always	28 (7.0)
Frequently	37 (9.2)
Sometimes	37 (9.2)
Never	298 (74.3)
Do not know	1 (0.3)

Results are expressed as *n* (%)

*TH* therapeutic hypothermia

**Fig. 1:**
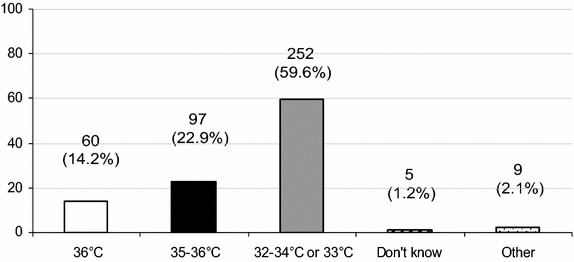
Distribution of the new targets temperature expressed as absolute number (percentage) after the Nielsen’s publication [6] (*n* = 423 respondents, expressed as percentage). Other targets (*n* = 9, 2.1 %) were documented as follows: 37 °C (*n* = 4, 0.9 %), 34 °C (*n* = 3, 0.7 %), and 35 °C (*n* = 2, 0.5 %)

**Fig. 2:**
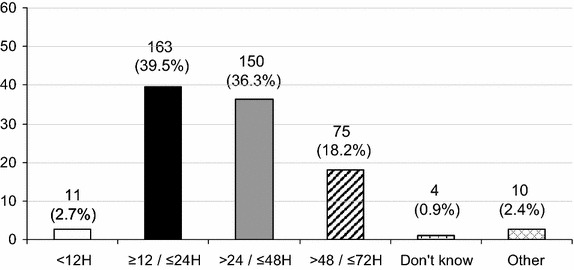
Distribution of the durations of the overall targeted temperature management period (*n* = 413 respondents, expressed as percentage). Other durations of the overall targeted temperature management phase were documented as follows: =72 h (2), >72 h (4) miscellaneous (4)

Since the TTM trial publication [6], 56 % of respondents declared no modification of their TTM practice, whereas 37 % declared a practical target temperature’s change (Additional file 2: Figure S4). This modification is applied in all CA patients in 52 % or in specific CA patients in 34 %, mainly in CA from cardiac origin (see details in Additional file 2: Figure S5). The new temperature targets presently preferred by respondents after the Nielsen’s trial are depicted in Fig. 1 [6]. The overall duration of the TTM after CA (i.e., including hypothermia whatever its level and/or normothermia) is now mainly applied between 12 and 24 h by 40 % of respondents, and between 24 and 48 h by 36 % (Fig. 2). Univariate analysis describing factors associated with modifications of the temperature level occurring after the TTM trial publication is described in Table 4.

**Table 4:** Comparison of respondents declaring a target temperature’s changes versus those without target temperature’s changes after the TTM study publication (univariate analysis)

	Respondents who changed their TT	Respondents who did not changed their TT	*P* value
University hospital	90/171 (52.6)	97/224 (43.3)	0.045
Public non-university hospital	65/171 (38.0)	115/224 (51.4)	
Private hospital	15/171 (8.8)	9/224 (4.0)	
Others	1/171 (0.6)	3/224 (1.3)	
Number of ICU beds	15.9 ± 7.9	15.1 ± 6.6	0.32
Number of residents and fellows	5.4 ± 5.9	4.5 ± 2.8	0.099
Number of full-time physicians	7.6 ± 4.7	6.9 ± 2.9	0.12
Use of a written CA protocol	77/170 (45.3)	102/224 (45.5)	0.87
Number of admissions <500/year	45/168 (26.8)	65/223 (29.1)	0.21
Number of admissions >500/year	123/168 (73.2)	158/223 (70.9)	
Number of CA admissions <30/year	98/172 (57.0)	126/227 (55.5)	0.87
Number of CA admissions >30/year	74/172 (43.0)	101/227 (44.5)	
Number of TH <30/year	108/154 (70.1)	129/212 (60.8)	0.22
Number of TH >30/year	46/154 (29.9)	83/212 (39.2)	
In-hospital cath-lab	124/172 (72.1)	161/227 (70.9)	0.87
ECLS use for refractory CA	92/172 (53.5)	101/227 (44.5)	0.07
Optimal ABP targeted	156/171 (91.2)	190/219 (86.8)	0.19
Specific TTM after CA^a^	166/171 (97.1)	213/227 (93.8)	0.008
No specific TTM after CA^a^	5/171 (2.9)	14/227 (6.1)	
TH after CA^b^	143/172 (83.1)	212/227 (93.4)	0.003
No TH after CA^b^	27/172 (15.7)	15/227 (6.6)	
TH duration			
<12 h	8/159 (5.0)	2/212 (0.9)	0.01
≥12 h/≤24 h	124/159 (80.0)	166/212 (78.3)	
>24 h/≤48 h	24/159 (15.1)	44/212 (20.8)	
>48 h	3/159 (1.9)	0/212 (0.0)	
Active rewarming	57/156 (36.5)	98/213 (46.0)	0.087
New TT			
32–34 °C	45/171 (26.3)	194/225 (86.2)	<0.0001
35–36 °C	78/171 (45.6)	15/225 (6.7)	
36 °C	44/171 (25.7)	11/225 (4.9)	
Other	4/171 (2.4)	5/225 (2.2)	
Overall TTM duration			
<12 h	7/169 (4.1)	3/220 (1.4)	0.074
≥12 h/≤24 h	75/169 (44.4)	81/220 (36.8)	
>24 h/≤48 h	61/169 (36.1)	83/220 (37.7)	
>48 h/≤72 h	24/169 (14.2)	42/220 (19.1)	
Other	2/169 (1.2)	11/220 (6.0)	

Results are expressed as *n* (%) unless expressed otherwise

*TTM* targeted temperature management, *TT* targeted temperature, *CA* cardiac arrest, *ECLS* extracorporeal life support, *ABP* arterial blood pressure, *TH* therapeutic hypothermia

^a^This item corresponds to the following question “In practice, do you generally pay a specific attention to temperature management in successfully resuscitated and unconscious CA patients”

^b^This item corresponds to the following question “In practice, do you generally use TH in successfully resuscitated and unconscious CA patients”

### General treatments along TTM

During the TTM period, patients were reported to always receive sedatives by 85 % of respondents (midazolam 83 % and/or propofol 40 %), analgesics by 78 % (sufentanil 64 % and fentanyl 23 %), and neuromuscular blockers by 47 % (cisatracurium 67 % and atracurium 29 %). A specific protocolized insulin treatment for glycemic control after CA was always used by 59 %. Fifty-six percent of respondents reported to never use a specific protocol regarding the PaO_2_ control, and 43 % to never use a specific protocol regarding the PaCO_2_ control. Additional results regarding the use of coronary angiography after the return of spontaneous circulation (ROSC), regarding the targeted arterial blood pressure after ROSC, regarding the initial cardiopulmonary resuscitation, regarding the use of brain computerized-tomography scanner, use of ventricular assist devices in refractory CA and in case of severe post-CA shock, and regarding prognostication after CA are described in the online supplementary files (Additional file 2: Figures S6, S7, S8, and S9; Tables S1, S2).

## Discussion

In this declarative survey, specific attention was generally paid by 94 % of respondents to TTM in unconscious and successfully resuscitated CA patients. The optimal target temperature to reach after CA remains unchanged for 56 % of respondents, whereas 37 % declare to target a new temperature following the TTM trial publication [6]. The current targets temperature are 32–34 °C (or 33 °C) in 60 %, 35–36 °C in 23 %, 36 °C in 14 %. TTM modifications were more frequently declared by physicians working in university hospitals. At present, TH is never induced using intravenous cold fluids by 38 % of respondents.

### TTM modifications

International guidelines recommended until recently the use of 32–34 °C-targeted TH in successfully resuscitated unconscious out-of-hospital adult CA patients, mainly for initial shockable rhythm [3]. However, the optimal level of the target temperature remains unknown. In the publication by Nielsen and co-workers, no main differences were observed between the two levels of target temperature: 33 °C (corresponding previously to TH) and 36 °C [6]. Considering debates after this large trial [7, 8, 21], ILCOR experts published a new recommendation regarding the TTM use after CA [9]. Indeed, since the results of the TTM publication, a significant proportion of physicians may shift back in their practice and abandon all sort of TTM after CA, and resume practice as mainly reported before the 2002 pivotal trials. Our survey confirms that roughly one-third of physicians decided to modify their current practice, mainly by using a smooth depth of TTM. However, most physicians seemed not to abandon the use of temperature management after CA, applying predominantly the previous recommended 33 °C target or a new 36 °C-TTM. Only a minority of physicians (6 %) herein declared to be unaware of any TTM after CA. This is concordant with a previous survey performed in France, observing a global 98 % adherence to recommendations regarding TTM [20]. Two-third of respondents declared in our survey not to have changed the target temperature, despite the TTM trial [6]. Among respondents who changed their target temperature, the majority decided to choose a TTM set around 36 °C, whereas some respondents decided to target the previous 32–34 °C range. One may hypothesize that the majority of respondents were not yet deeply convinced by the TTM trial or remained unaware of its publication, whereas some physicians possibly did not have previous protocol in their unit regarding the target temperature, or used a protocol targeting a temperature different from the recommended 32–34 °C range. Finally, our results seem in accordance with other studies describing that new scientific evidences resulting from recent randomized clinical trials are sometimes poorly implemented in clinical practice, as it has been shown for instance in the tight glycemic control area [22].

Interestingly, most of the respondents who changed their target temperature finally decided to choose preferentially an intermediate range between 35 and 36 °C, instead of the 36 °C precisely reported in the TTM trial. However, the 2015 ILCOR guidelines, recently published [23], recommend at present “TTM for adults with out-of-hospital CA with an initial shockable rhythm at a constant temperature between 32 and 36 °C for at least 24 h. Similar suggestions are made for out-of-hospital cardiac arrest with a non-shockable rhythm and in-hospital cardiac arrest.” In our survey, physicians changing their target temperature mostly applied these modifications for all CA patients, despite that the TTM trial was focused on out-of-hospital CA from cardiac origin. This suggests that physicians could use an adaptive TTM, since the TTM trial publication [6], mainly regarding the target temperature and the selection of patients that will receive TTM. However, these choices remain in accordance with these new ILCOR guidelines [23].

As expected in our study, the target temperature’s change was related to the TH/TTM characteristics performed by physicians (choice by physicians to perform TH/TTM in unconscious CA patients), and to the new target temperature. However, practical target temperature’s change seemed also related to the hospital category (i.e., university hospital or not). Interestingly, the TH/TTM durations seemed also associated to this temperature change: longer TTM durations seemed more often declared by physicians that did not change their practice. This could be paradoxical with the fact that the TTM trial preferentially used a prolonged duration of TTM as compared with the 2002 pivotal trials [1, 2, 6]. It can be argued that the respondents who changed their practice decided that TH should be no longer useful and consequently shortened the TH/TTM durations. However, prospective studies are warranted to confirm this issue.

### Modifications of the cooling methods

The majority of respondents herein declared to use basic external methods to implement TH. Close to our results, external methods were used in 54 % of cases in a previous survey conducted in France [20]. Similarly, other surveys observed that surface cooling and/or ice packs were mostly used in ICUs, usually reaching about 50–60 % [18, 19, 24, 25]. Our survey confirms that simple and less expensive methods, such as basic and external cooling, may represent the first choice for most physicians, despite that new arguments could modify this issue [26, 27].

Most of respondents declared in our survey that they never induce TH using intravenous cold fluids. In some countries where TH seemed underused (reaching 21.7, 43, and 55.1 % of cases), cold fluids as an induction method of cooling are also infrequently used (8.5, 7.4, and 22 % of respondents, respectively) [18, 25, 28]. Conversely, in United Kingdom with an 86 % use of TH, 71 % of respondents stated that TH was usually induced by the rapid infusion of cold fluids [19]. In Germany, cold packs and cold infusions were both equally used in about 60 %, mainly because of their lower cost [29]. In the study by Merchant and co-workers, non-United States respondents seemed more likely to cool with cold fluids as compared with United States respondents (36 versus 28 %, respectively), while ice packs were used in 40 and 60 %, respectively [30]. While 41 % of French ICUs previously declared to use cold fluid for TH induction [31], our survey observed that such cooling was presently not used in 38 % of cases. Indeed, two large randomized controlled studies using cold intravenous fluids to induce TH in the prehospital setting did not find improvement in outcome as compared with a standard TTM management, leading to important doubts regarding this specific cooling method for TH induction [32, 33]. This is also concordant with the new ILCOR guidelines that recently “recommends against prehospital cooling with rapid infusion of large volumes of cold intravenous fluid” [23]. However, whether “prehospital cooling using a rapid infusion of large volumes of cold intravenous fluid immediately after ROSC is not recommended, it may still be reasonable to infuse cold intravenous fluid where patients are well monitored and a lower target temperature (e.g., 33 °C) is the goal” [34].

### Other evaluated parameters along TTM

The previous published survey performed in France observed similar or close results to ours regarding associated treatments used during TTM [20]. However, we observed a slightly higher use of propofol (40 % presently versus 10 % in the previous study) [20]. This could be related to some recent publications showing a possible superiority regarding neurological prognostication by using short half-life sedatives [35]. Additionally, we found a possible lower percentage of use of neuromuscular blocking agents during TTM (47 % presently versus 97 % previously) [20]. To the best of our knowledge, our study is the first to evaluate the extremely low use of a specific protocol regarding PaO_2_ and PaCO_2_, despite the probable impact of such interventions [8, 36, 37]. This contrasts with glycemic control protocols that were here reported to be frequently used, suggesting that this parameter seems important for respondents as previously suggested in CA [38]. To the best of our knowledge, our survey is also the first to describe other TTM’s associated treatments of the post-CA period, such uses of coronary angiography and ventricular assist devices, and the arterial pressure target after CA along TTM (see the online supplement).

### Limitations

While offering new insights mainly regarding TTM implementation, our study has several limitations. First, the percentage of respondents is only 16 %, limiting results generalization. However, despite a median reported response rate reaching 63.3 % [39], lower response rates have also been described in other declarative surveys, between 2.9 and 18.1 % [40–42]. The usual response rates in the field of TH range from 13 to 98 %, with the lower limit roughly observed in our study [12, 15, 20, 24, 43, 44]. Second, as our survey is purely declarative, discrepancies may exist between our results and the real-life practice. We conducted this survey using the FICS website database including only physicians but not nurses. However, it is unlikely that other trained staff or nurses could apply different TTM prescriptions as compared with those declared in the present survey. Third, it could be argued that our survey only concerns intensivists working in France. However, the FICS includes several responders from numerous French-speaking countries, herein reaching 13 % of respondents of this survey. Because only French-speaking physicians were here able to respond, our results can mainly be applied for French-speaking countries. Considering the high rate (98 %) of TH implementation in France as previously published [20], our result regarding the percentage of TH changes induced by the TTM publication seems likely. Finally, our survey collected answers from some responders working in the same ICU that could lead to bias. However, 264 ICUs from 11 countries were involved in the present survey, showing a large representative response to correctly evaluate the TTM and cooling changes.

## Conclusion

The TTM trial publication showing no main difference between 36 and 33 °C after CA has induced a modification of current practices in one-third of respondents. However, the 32–34 °C target temperature remained unchanged for 56 % of respondents. Prospective epidemiologic studies may evaluate the impact on patients’ outcome of those practices. Educational actions are needed to promote better knowledge translations of trial results and guidelines into clinical practice.

## Additional files

10.1186/s13613-015-0104-6 French questionnaire.
10.1186/s13613-015-0104-6 Additional Figures S1–S9 and Tables S1–S2.

## Abbreviations

CA: cardiac arrest
FICS/*SRLF*: French Intensive Care Society (société de réanimation de langue française)
ICU: intensive care unit
ILCOR: international liaison committee on resuscitation
TH: therapeutic hypothermia
TTM: targeted temperature management
ROSC: return of spontaneous circulation
