# Supplementary material for: Changes in cardiac arrest patients’ temperature management after the 2013 “TTM” trial: results from an international survey
Source: Ann Intensive Care. 2016 Jan 12;6:4. doi: 10.1186/s13613-015-0104-6 (PMC4709360; doi:10.1186/s13613-015-0104-6)
Supplement: Supplementary file 1 — 10.1186/s13613-015-0104-6 French questionnaire. [file 13613_2015_104_MOESM1_ESM.pdf]

A. DONNEES GENERALES DU SERVICE REPONDEUR

*1. Lieu d'exercice:

Ville*:

Pays*:

Code postal*:

Hôpital*:

Service*:

Nom du Chef de service*:

Adresse e-mail:

Téléphone:

2. Type d'hôpital:

- Universitaire
- Public non universitaire
- PSPH
- Privé
- Autre (veuillez préciser)

3. Nombre de lits dans le service:

Lits de réanimation:

Lits de surveillance continue:

Nombre total, si lits indifférenciés:

4. Nombre de médecins dans le service:

Séniors (PU-PH, PH, CCA, PAC … A exprimer en équivalent temps plein)

Internes

5. Nombre d'admissions approximatif dans le service en 2013:

- <300
- 300 à 500
- 500 à 800
- >800

***6. Quel a été le nombre approximatif d’admissions pour arrêt cardiaque (AC), intra ou extra hospitalier, dans votre service d'exercice (principal si plusieurs) en 2013 ?**

- <10
- 10 à 20
- 20 à 30
- 30 à 40
- 40 à 50
- >50

***7. Y a-t-il dans votre hôpital une unité de cardiologie interventionnelle ?**

- Oui
- Non

***8. Si oui, est elle disponible 24 heures/24, 7 jours/7 ?**

- Oui
- Non

9. Dans votre service, utilisez-vous une Procédure Opérationnelle Standardisée concernant l’AC ?

- Oui
- Non
- Ne sais pas

B. PRISE EN CHARGE GENERALE DE L'AC

***10. Dans votre pratique, quelle durée considérez-vous comme généralement suffisante pour arrêter la Réanimation Cardio-Pulmonaire spécialisée?**

- [ ] 20 minutes
- [ ] 30 minutes
- [ ] Ne sais pas
- [ ] Autre (veuillez préciser, en minutes)

11. Après Reprise d'une Activité Circulatoire Spontanée (RACS), en dehors des étiologies extra-cardiaques évidentes ou d'un sus-décalage patent du segment ST à l'ECG, faites-vous réaliser une coronarographie ?

○ Toujours ○ Fréquemment ○ Parfois ○ Jamais ○ Ne sais pas

12. Après RACS (en dehors des étiologies cardiaques prouvées), faites vous réaliser un scanner cérébral initial ?

○ Toujours ○ Fréquemment ○ Parfois ○ Jamais ○ Ne sais pas

13. Après RACS, avez vous un objectif précis de Pression Artérielle (PA) cible ?

○ Oui ○ Non ○ Ne sais pas

14. Si oui, quel(s) objectif(s) de PA, moyenne (M) ou systolique (S) visez-vous ?

- [ ] PAS < 90 mm Hg
- [ ] PAS = 90 mm Hg
- [ ] PAS > 90 mm Hg
- [ ] PAM ≥ 65 mm Hg
- [ ] PAM > 70 mm Hg
- [ ] Autre (veuillez préciser, S ou M en mm Hg)

***15. Dans votre pratique, utilisez-vous l'ECMO artério-veineuse dans certain(s) cas d'AC réfractaire(s) ?**

○ Oui ○ Non ○ Ne sais pas

16. Si oui, utilisez-vous l'ECMO artério-veineuse dans certains cas d'AC réfractaire par hypothermie accidentelle ?

○ Toujours ○ Fréquemment ○ Parfois ○ Jamais ○ Ne sais pas

17. Si oui, utilisez-vous l'ECMO artério-veineuse en cas d'AC réfractaire dans certaines intoxications médicamenteuses ?

- ○ Toujours
- ○ Fréquemment
- ○ Parfois
- ○ Jamais
- ○ Ne sais pas

18. Si oui, utilisez-vous l'ECMO artério-veineuse en cas d'AC réfractaire dans certain(s) cas d'infarctus du myocarde à la phase aiguë ?

- ○ Toujours
- ○ Fréquemment
- ○ Parfois
- ○ Jamais
- ○ Ne sais pas

19. Si oui, utilisez-vous l'ECMO artério-veineuse en cas d'AC réfractaire au cours de certain(s) cas de trouble(s) du rythme ventriculaire réfractaire(s) ?

- ○ Toujours
- ○ Fréquemment
- ○ Parfois
- ○ Jamais
- ○ Ne sais pas

20. Dans votre pratique, utilisez-vous parfois ou souvent un dispositif d'assistance cardio-circulatoire en cas de choc sévère à réfractaire après AC récupéré avec succès (RACS stable) ?

- ○ Oui
- ○ Non
- ○ Ne sais pas

21. Si oui (plusieurs réponses possibles) ?

- ☐ ECMO
- ☐ Assistance intra-ventriculaire type Impella
- ☐ CPIA
- ☐ Autre (veuillez préciser)

C. CONTROLE THERMIQUE (NORMOTHERMIE ET HYPOTHERMIE THERAPEUTIQUE) & TRAITEMENTS ASSOCIES

***22. Dans votre pratique, comment surveillez-vous généralement la température chez un patient récupéré (RACS) après AC ?**

- ☐ Mesure externe (tympanique ou axillaire)
- ☐ Sonde rectale
- ☐ Sonde œsophagienne
- ☐ Sonde vésicale
- ☐ Endovasculaire (Swan-Ganz, autre technique de thermodilution)
- ☐ Aucune mesure thermique spécifique à ces patients
- ☐ Autre (veuillez préciser)

***23. Dans votre pratique, est-t-il généralement réalisé une gestion spécifique de la température (contrôle thermique par lutte contre l'hyperthermie, normothermie ...) chez un patient inconscient après AC ?**

○ Oui ○ Non ○ Ne sais pas

24. Si non, pourquoi ?

- ☐ Etudes non convaincantes : pas assez nombreuses ou discordantes
- ☐ Mauvaise connaissance des données de la littérature médicale
- ☐ Techniquement trop difficile à mettre en place
- ☐ Craintes des complications du contrôle thermique
- ☐ Autre (veuillez préciser)

***25. Dans votre pratique, est-t-il généralement réalisé une hypothermie thérapeutique (HT) chez un patient inconscient après AC ?**

○ Oui ○ Non ○ Ne sais pas

26. Si non, pourquoi ?
(Atteindre alors la question 43)

- ☐ Etudes non convaincantes : pas assez nombreuses ou discordantes
- ☐ Méconnaissance des données de la littérature médicale
- ☐ Techniquement trop difficile à mettre en place
- ☐ Craintes des complications de l'hypothermie
- ☐ Autre (veuillez préciser)

27. Si oui quel est le nombre approximatif d'HT pour AC pratiquées en 2013 dans votre pratique ?

- ○ <10
- ○ 10 à 20
- ○ 20 à 30
- ○ 30 à 40
- ○ 40 à 50
- ○ >50

28. Chez un patient toujours comateux (ne répondant pas aux ordres simples) après AC
extra-hospitalier récupéré, réalisez-vous l'HT en cas de FV/TV sans pouls ?

○ Toujours ○ Souvent ○ Parfois ○ Jamais ○ Ne sais pas

29. Chez un patient toujours comateux (ne répondant pas aux ordres simples) après AC
extra-hospitalier récupéré, réalisez-vous l'HT en cas d'asystolie/dissociation électro-mécanique ?

○ Toujours ○ Souvent ○ Parfois ○ Jamais ○ Ne sais pas

30. Chez un patient toujours comateux (ne répondant pas aux ordres simples) en cas d'AC
intra-hospitalier récupéré, réalisez-vous l'HT en cas de FV/TV sans pouls ?

○ Toujours ○ Souvent ○ Parfois ○ Jamais ○ Ne sais pas

31. Chez un patient toujours comateux (ne répondant pas aux ordres simples) en cas d'AC
intra-hospitalier récupéré, réalisez vous l'HT en cas d'asystolie/dissociation électro-mécanique ?

○ Toujours ○ Fréquemment ○ Parfois ○ Jamais ○ Ne sais pas

32. Induisez-vous l'HT par perfusion de solutés froids ?

- Toujours
- Souvent
- Parfois
- Jamais
- Ne sais pas

33. Maintenez-vous l'HT par perfusion de solutés froids ?

- Toujours
- Fréquemment
- Parfois
- Jamais
- Ne sais pas

34. Induisez-vous ou maintenez-vous l'HT par méhode conventionnelle avec ventilateur, packs ou vessie de glace, ou linges mouillés ?

- Toujours
- Souvent
- Parfois
- Jamais
- Ne sais pas

35. Induisez-vous ou maintenez-vous l'HT par refroidissement de surface avec circulation d'eau glacée (« water blanket ») ?

- Toujours
- Souvent
- Parfois
- Jamais
- Ne sais pas

36. Induisez-vous ou maintenez-vous l'HT par refroidissement de surface avec circulation d'air froid (« air blanket ») ?

- Toujours
- Souvent
- Parfois
- Jamais
- Ne sais pas

37. Induisez-vous ou maintenez-vous l'HT par systèmes adhésifs avec gel échangeurs de chaleur et système à eau circulante ?

- Toujours
- Souvent
- Parfois
- Jamais
- Ne sais pas

38. Induisez-vous ou maintenez-vous l'HT par dispositif endovasculaire ?

- Toujours
- Souvent
- Parfois
- Jamais
- Ne sais pas

39. Aucun de ces moyens ?

- [ ] Oui
- [ ] Ne sais pas
- [ ] Autre (veuillez préciser)

40. Dans votre pratique, pendant combien de temps l'HT à 33°C ou entre 32-34°C est-elle généralement maintenue ?

- <12 h
- 12 h-24 h
- 24 h-48 h
- >48h

41. Dans votre pratique, en fin d'HT, réalisez-vous généralement un réchauffement (plusieurs réponses possibles) ?

- [ ] Actif
- [ ] Passif
- [ ] Ne sais pas

42. Si actif, quelle vitesse de réchauffement usuelle visez-vous ?

- 0,1°C/heure
- 0,3°C/heure
- 0,5°C/heure
- > 0,5 °C/heure
- Autre (veuillez préciser)

***43. Avez-vous modifié l'objectif de température depuis la dernière publication de décembre 2013 concernant le niveau cible de contrôle thermique (Targeted temperature management at 33°C versus 36°C after cardiac arrest. Nielsen N, Wetterslev J, Cronberg T, Erlinge D, Gasche Y, Hassager C, Horn J, Hovdenes J, Kjaergaard J, Kuiper M, Pellis T, Stammet P, Wanscher M, Wise MP, Åneman A, Al-Subaie N, Boesgaard S, Bro-Jeppesen J, Brunetti I, Bugge JF, Hingston CD, Juffermans NP, Koopmans M, Køber L, Langørgen J, Lilja G, Møller JE, Rundgren M, Rylander C, Smid O, Werer C, Winkel P, Friberg H; TTM Trial Investigators. N Engl J Med. 2013 Dec 5;369(23):2197-206) ?**

- Oui
- Non
- Ne sais pas

44. Si oui, dans quels cas ?

- Tous les AC
- AC ciblés
- Ne sais pas

45. Si AC ciblés, lesquels ?

- AC d'origine présumée cardiaque
- AC par trouble du rythme
- Autre (veuillez préciser)

***46. Quel est votre objectif actuel de température pendant la phase de contrôle thermique ?**

- 36°C
- 35-36°C
- 32-34°C (ou 33°C)
- Ne sais pas
- Autre (veuillez préciser)

47. Dans votre pratique, pendant combien de temps au total le contrôle thermique (incluant hypothermie et/ou normothermie) est-il en général maintenu ?

- <12 heures
- ≤ 12 heures-≤ 24 heures
- > 24 heures-≤ 48 heures
- > 48 heures-≤ 72 heures
- Ne sais pas
- Autre (veuillez préciser)

***48. Pendant la phase de contrôle thermique (et/ou d'hypothermie), vos patients sont-ils sédatés ?**

- Toujours
- Fréquemment
- Parfois
- Jamais
- Ne sais pas

49. Si oui, quel(s) hypnotique(s) utilisez-vous ?

- Midazolam
- Propofol
- Ne sais pas
- Autre (veuillez préciser)

***50. Pendant la phase initiale de contrôle thermique (et/ou d'hypothermie), vos patients sont-ils analgésiés ?**

- Toujours
- Fréquemment
- Parfois
- Jamais
- Ne sais pas

51. Si oui, quel(s) analgésique(s) utilisez vous ?

- Morphine
- Fentanyl
- Sufentanil
- Remifentanil
- Ne sais pas

Autre (veuillez préciser)

***52. Pendant la phase initiale de contrôle thermique (et/ou d'hypothermie), vos patients sont-ils curarisés ?**

- Toujours
- Fréquemment
- Parfois
- Jamais
- Ne sais pas

53. Si oui, quel(s) curare(s) utilisez vous ?

- ☐ Atracurium
- ☐ Cisatracurium
- ☐ Rocuronium
- ☐ Vecuronium
- ☐ Ne sais pas
- ☐ Autre (veuillez préciser)

***54. Utilisez-vous un protocole spécifique de gestion de la glycémie par l'insuline, après AC ?**

○ Toujours ○ Fréquemment ○ Parfois ○ Jamais ○ Ne sais pas

55. Si oui, quel objectif visez vous (en mmol/L) ?

***56. Utilisez-vous un protocole spécifique de gestion de la PaO2, après AC ?**

○ Toujours ○ Fréquemment ○ Parfois ○ Jamais ○ Ne sais pas

57. Si oui, quel objectif visez vous (en mm Hg) ?

***58. Utilisez-vous un protocole spécifique de gestion de la PaCO2, après AC ?**

○ Toujours ○ Fréquemment ○ Parfois ○ Jamais ○ Ne sais pas

59. Si oui, quel objectif visez vous (en mm Hg) ?

D. EVALUATION PRONOSTIQUE

***60. En général, faites-vous réaliser un Electro-Encéphalo-Gramme (EEG) ?**

- ○ Toujours
- ○ Fréquemment
- ○ Parfois
- ○ Jamais
- ○ Ne sais pas

61. Si oui, en phase d'hypothermie ?

- ○ Toujours
- ○ Fréquemment
- ○ Parfois
- ○ Jamais
- ○ Ne sais pas

62. Si oui après le réchauffement et/ou dès la normothermie ?

- ○ Toujours
- ○ Fréquemment
- ○ Parfois
- ○ Jamais
- ○ Ne sais pas

63. En cas de troubles de la conscience persistants, la fréquence de réalisation est plutôt ?

- ☐ Pluriquotidienne
- ☐ Quotidienne
- ☐ Tous les 2 jours
- ☐ Continue (ou quantitative)
- ☐ Ne sais pas
- ☐ Autre (veuillez préciser)

64. Concernant les facteurs pronostiques après AC, utilisez vous un ou des critères "cliniques" ?

- ☐ Présence d'un témoin de l'AC
- ☐ Durée de réanimation (« no flow », « low flow »)
- ☐ Absence de réflexes du tronc cérébral (reflexe cornéen, ventilation…)
- ☐ Absence de réactivité pupillaire
- ☐ Convulsion(s) clinique(s)
- ☐ Score de Glasgow
- ☐ Score moteur du Glasgow
- ☐ Four score
- ☐ Myoclonies précoces
- ☐ Aucun
- ☐ Ne sais pas
- ☐ Autre (veuillez préciser)

65. Concernant les facteurs pronostiques après AC, utilisez-vous un ou des critères biologiques ?

- ☐ Lactatémie
- ☐ Neuron Specific Enolase
- ☐ Proteine S100B
- ☐ Aucun
- ☐ Ne sais pas
- ☐ Autre (veuillez préciser)

66. Concernant les facteurs pronostiques après AC, utilisez-vous un ou des critères EEG ?

- ☐ EEG "plat"
- ☐ Pointe(s)-onde(s)
- ☐ "Burst-suppression"
- ☐ Rythme alpha
- ☐ Absence de réactivité
- ☐ EEG continu ou quantitatif de mauvais pronostic
- ☐ BIS ou autre système équivalent de mauvais pronostic
- ☐ Aucun
- ☐ Ne sais pas
- ☐ Autre (veuillez préciser)

67. Concernant les facteurs pronostiques après AC, utilisez-vous les Potentiel(s) Evoqué(s) ?

☐ Somesthésiques ☐ Auditifs ☐ Aucun ☐ Ne sais pas

68. Concernant les facteurs pronostiques après AC, utilisez-vous les données d'une TDM cérébrale ?

○ Toujours ○ Fréquemment ○ Parfois ○ Jamais ○ Ne sais pas

69. Concernant les facteurs pronostiques après AC, utilisez-vous les données d'une IRM cérébrale ?

○ Toujours ○ Fréquemment ○ Parfois ○ Jamais ○ Ne sais pas

70. Si oui, utilisez vous une IRM fonctionnelle ?

- Toujours
- Fréquemment
- Parfois
- Jamais
- Ne sais pas

71. Concernant les facteurs pronostiques après AC utilisez-vous parmi ceux précédemment cités ?

- Aucun en particulier
- Une combinaison de plusieurs des critères cités ci-dessus (> 3)
- Ne sais pas
- Autre (veuillez préciser)

***72. Concernant l'évolution du patient, utilisez-vous une Procédure Opérationnelle Standardisée concernant une possible Limitation des Thérapeutiques (LAT) ?**

- Oui
- Non
- Ne sais pas
- Autre (veuillez préciser)

73. Si oui (plusieurs réponses possibles):

- Avis collégial intra-service consigné
- Avis extérieur consigné
- Autre (veuillez préciser)

***74. A quel moment réalisez-vous en général une première décision de LAT ?**

-
- J3-J7
- J7-J14
- Après J15
- Autre (veuillez préciser)
