# Supplementary material for: Changes in cardiac arrest patients’ temperature management after the 2013 “TTM” trial: results from an international survey
Source: Ann Intensive Care. 2016 Jan 12;6:4. doi: 10.1186/s13613-015-0104-6 (PMC4709360; doi:10.1186/s13613-015-0104-6)
Supplement: Supplementary file 2 — 10.1186/s13613-015-0104-6 Additional Figures S1–S9 and Tables S1–S2. [file 13613_2015_104_MOESM2_ESM.doc]

**CHANGES IN CARDIAC ARREST PATIENTS' TEMPERATURE MANAGEMENT AFTER THE 2013 “*TTM*” TRIAL: RESULTS FROM AN INTERNATIONAL SURVEY**

**Online additional data: 9 figures, 2 tables.**

**Additional** **Figure S1.** Distributive number of respondents over time (n=518).

**Additional** **Figure S2.** Reason(s) why therapeutic hypothermia is not considered in unconscious cardiac arrest patients (N=41 responders; 51 answers expressed as percentage of responses).

**Additional** **Figure S3.** Site(s) of temperature monitoring after the return of spontaneous circulation in cardiac arrest patients (N=423 respondents; 688 answers expressed as percentage of response).

External measure: skin or epitympanic measurements; Intravascular: Swan-Ganz catheter or PiCCO® measurements; No specific: no specific temperature measurement.

**Additional** **Figure S4.** Modification of the target temperature since the Nielsen’s publication (n=423 respondents).

“?” denotes the answer “Do not know”.

**Additional** **Figure S5.** Characteristics of cardiac arrest or main reasons for which TTM modifications were applied, “if the TTM have changed for some specific cardiac arrest patients only since the Nielsen’s trial publication” (N=83 responders; 108 answers expressed as percentage of responses).

TTM: targeted temperature management. Specific answers regarding the “Potential TH side-effects” were documented as follows: bleeding, hemodynamic unstability, shock.

**Additional** **Figure S6.** Number of respondents performing a coronary angiogram after the return of spontaneous circulation in cardiac arrest patients without an obvious extra-cardiac cause and significant ST elevation myocardial infarction on electrocardiogram (n=452 respondents).

**Additional** **Figure S7.** Optimal arterial blood pressure targeted after the return of spontaneous circulation in cardiac arrest patients (n=405 answers).

An optimal arterial blood pressure was targeted by 87% of respondents, mainly using a mean arterial pressure target ≥ 65 mmHg (56.5%).

SABP: systolic arterial blood pressure; MABP: mean arterial blood pressure.

**Additional** **Figure S8.** Duration of advanced cardiopulmonary resuscitation considered as generally sufficient by respondents before stopping resuscitation (n=470 responses expressed as percentage).

**Additional** **Figure S9.** Number of respondents performing initially a brain computerized-tomography scanner after return of spontaneous circulation, in patients without an obvious cardiac cause of cardiac arrest (n=452).

**Additional** **Table S1.** Indications of Extra-Corporeal Life Support implementation in case of refractory cardiac arrest (N respondents).

Accidental hypothermia (N=268)

Always 48 (18)

Frequently 43 (16)

Sometimes 61 (23)

Never 74 (28)

Do not know 42 (15)

Drug poisoning (N=264)

Always 52 (20)

Frequently 59 (22)

Sometimes 84 (32)

Never 48 (18)

Do not know 21 (8)

Acute myocardial infarction (N=265)

Always 23 (9)

Frequently 41 (15)

Sometimes 100 (38)

Never 69 (26)

Do not know 32 (12)

Malignant arrhythmia (N=266)

Always 21 (8)

Frequently 39 (15)

Sometimes 88 (33)

Never 78 (29)

Do not know 40 (15)

Results are expressed as n (%). Forty-two per cent of respondents declared to use arterio-venous extracorporeal life support in some refractory CA patients, whereas 56% were not.

Fifty-three per cent of respondents declared to use ventricular assist devices in case of severe or refractory post-CA shock after achieving sustained return of spontaneous circulation, using arterio-venous extracorporeal life support (ECLS) in 73%, intra-aortic balloon pump in 65%, and Impella^TM^ in 14%; 42% of respondents declared not to use any ventricular assist devices in such patients.

**Additional** **Table S2.** Main clinical, biological, electrophysiological, and radiographic parameters reported to be used for prognostication after cardiac arrest (N respondents).

Clinical tools (N=399)

Absence of bystander CPR 243 (61)

Time from collapse to first CPR and CPR duration 351 (88)

Absence of brainstem reflexes 379 (95)

Fixed pupils 310 (78)

Seizure / status epilepticus 287 (72)

Myoclonia (in the early post-CA phase) 295 (74)

Glasgow coma score 165 (41)

Motor response of the Glasgow coma score 118 (30)

Four score 22 (6)

Biological tools (N=389)

Lactate 157 (40)

Neuron specific enolase 117 (30)

S100B protein 25 (6)

Other biomarkers (creatinine...) 7 (2)

No biomarker use 141 (36)

EEG or equivalent (N=392)*

Flat EEG 300 (77)

"Burst-suppression" 199 (51)

Absence of reactivity 197 (50)

Spike wave(s) 116 (30)

Alpha rhythm 46 (51)

Continuous or quantitative 41 (10)

Bispectral monitoring (BIS^TM^) 25 (6)

No EEG 31 (8)

Other electrophysiological tools (N=390)

Somato-sensory evoked potential (SSEP) 189 (48)

Auditory evoked potential 72 (18)

No evoked potential 174 (45)

Brain CT-scan (N=393)

Always 20 (5)

Frequently 69 (18)

Sometimes 165 (42)

Never 134 (34)

MRI (N=396)

Always 11 (3)

Frequently 61 (15)

Sometimes 189 (48)

Never 128 (32)

Results are expressed as n (%). CT: computer-tomography. MRI: Magnetic Resonance Imagery. Electro-encephalogram: EEG. *: EEG is frequently performed according to 39% of respondents: mostly never performed during the TH period (65%), and always performed after the rewarming phase and/or as soon as normothermia is reached (41%). A combination of more than three prognostic factors (within clinical and biological tools, EEG and electrophysiological tests, brain CT scan, and MRI) is used by 89% of respondents.

A protocol of withdrawal of life sustaining treatment was reported to be available by 79% of respondents. The first decision for treatments withdrawal is generally performed between day 3 to day 7 by 60% of respondents, and between day 7 to day 14 by 28%.
